# Specialist consultation activity and costs in Australia: Before and after the introduction of COVID-19 telehealth funding

**DOI:** 10.1177/1357633X211042433

**Published:** 2021-11-02

**Authors:** Keshia R De Guzman, Liam J Caffery, Anthony C Smith, Centaine L Snoswell

**Affiliations:** 1 Centre for Online Health, 430948 The University of Queensland, Brisbane, Australia; 2 Centre for Health Services Research, 558219 The University of Queensland, Brisbane, Australia; 3 Centre for Innovative Medical Technology, 1974 University of Southern Denmark, Denmark

**Keywords:** Medicare Benefits Schedule, telehealth, telemedicine, funding, specialist, COVID-19, pandemic

## Abstract

This study describes and analyses the Medicare Benefits Schedule (MBS) activity and cost data for specialist consultations in Australia, as a result of the coronavirus disease 2019 (COVID-19) pandemic. To achieve this, activity and cost data for MBS specialist consultations conducted from March 2019 to February 2021 were analysed month-to-month. MBS data for in-person, videoconference and telephone consultations were compared before and after the introduction of COVID-19 MBS telehealth funding in March 2020. The total number of MBS specialist consultations claimed per month did not differ significantly before and after the onset of COVID-19 (*p* = 0.717), demonstrating telehealth substitution of in-person care. After the introduction of COVID-19 telehealth funding, the average number of monthly telehealth consultations increased (*p* < 0.0001), representing an average of 19% of monthly consultations. A higher proportion of consultations were provided by telephone when compared to services delivered by video. Patient-end services did not increase after the onset of COVID-19, signifying a divergence from the historical service delivery model. Overall, MBS costs for specialist consultations did not vary significantly after introducing COVID-19 telehealth funding (*p* = 0.589). Telehealth consultations dramatically increased during COVID-19 and patients continued to receive specialist care. After the onset of COVID-19, the cost per telehealth specialist consultation was reduced, resulting in increased cost efficiency to the MBS.

## Introduction

In Australia, funding through the Medicare Benefits Schedule (MBS) for telehealth specialist consultations has existed since 2011.^1,2^ However, this funding was subject to strict eligibility criteria; patients had to reside outside of major cities (geographical limits) and specialist consultations needed to be provided through videoconferencing.^2,3^ To incentivise specialists to conduct video consultations, with an aim to increase access for rural and remote patients, telehealth reimbursement was set at 150% of comparable in-person consultation MBS rebates. Telehealth consultations were also eligible for concurrent patient-end reimbursement claims, which is a small payment for a clinician local to the patient (e.g. general practitioner (GP) or nurse) to join the consultation and provide support and technical assistance.^4^ In doing this, primary care clinicians could access specialist information provided to their patients in real-time during the telehealth consultation, improving the continuity of patient care.^4^ Although reimbursement incentives and MBS funding have been available for specialist telehealth consultations for a decade, telehealth uptake by specialists in Australia has remained relatively low.^2,5^ This changed in March 2020, during the coronavirus disease 2019 (COVID-19) pandemic, when eligibility criteria were relaxed and telehealth use increased dramatically.

At the onset of COVID-19, in March 2020, the Australian government introduced new MBS funding so that specialists (and other clinicians) could provide telehealth consultations.^3,6,7^ Compared to the pre-existing funding, access to COVID-19 MBS telehealth funding was not geographically restricted.^3,6^ MBS reimbursement of specialist telehealth consultations was further extended to include telephone consultations in addition to video consultations.^6^ This expansion of telehealth coverage enabled specialists to continue to provide care during COVID-19, while simultaneously reducing potential disease transmission.^8–11^ As a result, COVID-19 was a catalyst for the sudden shift from in-person to telehealth specialist care.^7,12^ Under the COVID-19 telehealth funding, MBS reimbursement for video consultations was at parity payment with comparable in-person consultations. Given this change in reimbursement value, it is important to assess whether the introduction of COVID-19 telehealth funding resulted in an efficient allocation of MBS resources. Due to the rapid uptake of telehealth during COVID-19, there is also a need to observe how the shift towards telehealth affected specialist care in Australia. This study aimed to describe and analyse the MBS activity data and associated costs for specialist consultations in Australia before and after the onset of COVID-19. These findings will help inform future telehealth policy decisions and further the development of alternative models of specialist care.

## Methods

This study was a retrospective analysis of MBS-subsidised specialist consultations conducted in Australia from March 2019 to February 2021, covering a year before and a year after the introduction of COVID-19 telehealth funding (which occurred in March 2020). Telehealth activity and cost data were obtained from publicly available MBS data.^13^ MBS specialist activity and cost data for in-person, video conference and telephone consultations were collated monthly for the 24-month time period. Specialist activity and associated costs, before and after the introduction of COVID-19 telehealth funding, were analysed by comparing the average number of MBS specialist consultations and costs per month for pre-COVID-19 (March 2019 to February 2020) and post-COVID-19 (March 2020 to February 2021) time periods. The average proportion (%) of monthly specialist consultations and costs delivered via telehealth and in-person modalities was also reported. Paired *t*-tests were used to determine if the average number of specialist consultations and costs per month differed significantly after the onset of COVID-19. A level of *p* < 0.05 was considered statistically significant. When analysing MBS data, patient-end services were not counted as specialist telehealth consultations. However, patient-end service claims were added to the total MBS costs as they contribute to the overall costs associated with specialist telehealth consultations. This study received notification of ethics exemption from The University of Queensland, Human Research Ethics Review (2021/HE001216).

## Results

### Overall specialist activity

**Figure 1.**
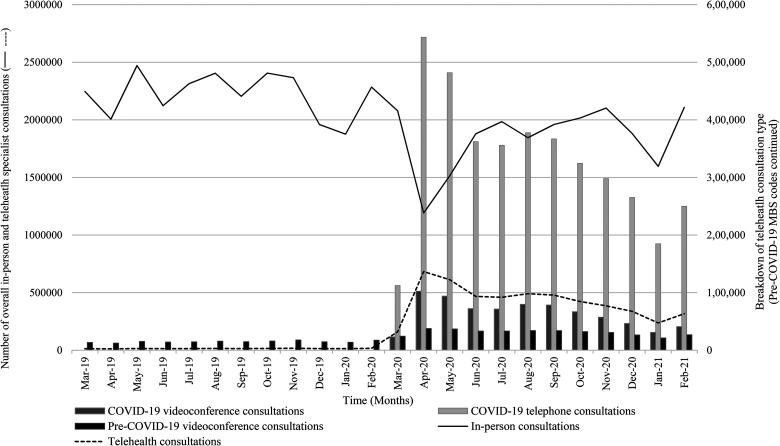
Medicare Benefits Schedule (MBS) activity for specialist in-person and telehealth consultation codes from March 2019 to February 2021.

The overall MBS activity for specialist in-person and telehealth consultations in Australia from March 2019 to February 2021 is shown in Figure 1. The total number of MBS specialist consultations claimed from March 2019 to February 2020 was, on average, 2.2 million per month, compared to 2.3 million per month from March 2020 to February 2021 (Table 1). Although this represented a minimal change (∼1%) in overall activity, there was no significant change in the average monthly number of specialist consultations after the COVID-19 MBS telehealth funding changes were introduced (*p* = 0.717).

**Table 1.** Comparison of MBS activity and associated costs for specialist consultations and patient-end services provided before and after the onset of the COVID-19 pandemic.

	Time period		
Specialist activity	Pre-COVID-19 period Mar 2019 to Feb 2020	Post-COVID-19 period Mar 2020 to Feb 2021	*P*-value
	Average number of MBS specialist consultations provided per month, *N* (%)		
Total consultations	2,236,900 (100.0)	2,268,073 (100.0)	0.717
In-person consultations	2,222,025 (99.3)	1,846,794 (81.2)	0.001
Telehealth consultations	14,875 (0.7)	421,279 (18.8)	<0.0001
Pre-COVID-19 videoconference	14,875 (0.7)	30,810 (1.4)	
COVID-19 videoconference	Not applicable	63,536 (2.8)	
COVID-19 telephone	Not applicable	326,933 (14.6)	
Patient-end services	5924 (0.3)	6497 (0.3)	0.061
	Average costs to the MBS for specialist consultations provided per month, AU$ (%)		
Total consultations	176,028,067 (100.0)	180,455,237 (100.0)	0.589
In-person consultations	172,521,216 (98.0)	144,533,797 (79.8)	0.004
Telehealth consultations	3,052,661 (1.7)	35,427,581 (19.9)	<0.0001
Pre-COVID-19 videoconference	3,052,661 (1.7)	5,987,359 (3.4)	
COVID-19 videoconference	Not applicable	5,715,683 (3.2)	
COVID-19 telephone	Not applicable	23,724,538 (13.4)	
Patient-end services	454,190 (0.3)	493,859 (0.3)	0.096

MBS: Medicare Benefits Schedule; COVID-19: coronavirus disease 2019.

### Telehealth specialist activity and patient-end services

Prior to COVID-19, specialist consultations were primarily delivered in-person with a monthly average of 0.7% of video consultations (Table 1). After the COVID-19 telehealth funding changes were introduced, there was an immediate increase in monthly telehealth delivery. In the post-COVID-19 time period, telehealth consultations (telephone and videoconference) represented an average of 19% of monthly specialist consultations. The dramatic increase in the average number of monthly telehealth consultations in the post-COVID-19 period compared to the pre-COVID-19 period was significant (*p* < 0.0001). Similarly, the average number of monthly in-person consultations significantly decreased after the onset of COVID-19, demonstrating the increase in telehealth substitution (*p* = 0.001). Telehealth consultations reached a peak in April 2020, accounting for 36% of all specialist consultations (Figure 1). There was a small increase in patient-end services after the onset of COVID-19, however, this increase was not significant, and not in line with the overall increase in telehealth activity (*p* = 0.061).

### Telephone and videoconference specialist consultations

Prior to COVID-19, only video consultations were eligible for MBS reimbursement. However, after the introduction of telephone reimbursement at the onset of COVID-19, telephone use dominated videoconference use in the months following March 2020 (Figure 1). In the post-COVID-19 period, telephone accounted for 15% of specialist consultations while video consultations accounted for 4% (Table 1). Of the average number of telehealth consultations (421,279) delivered per month after the onset of COVID-19, 78% (326,933) were telephone consultations.

### Overall specialist costs to the MBS

**Figure 2.**
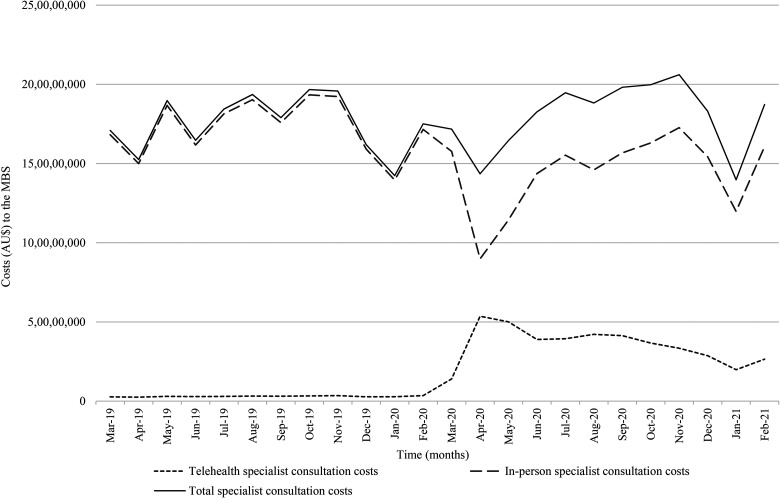
Costs to the Medicare Benefits Schedule (MBS) for specialist in-person and telehealth consultations from March 2019 to February 2021.

The overall costs to the MBS for specialist in-person and telehealth consultations in Australia from March 2019 to February 2021 are shown in Figure 2. Costs for specialist consultations did not change after the introduction of COVID-19 MBS telehealth funding changes (Table 1). The average monthly costs to the MBS for specialist consultations did not differ between the pre-COVID-19 and post-COVID-19 time periods (*p* = 0.589). The average monthly cost to the MBS associated with patient-end services did not differ significantly after the onset of COVID-19, demonstrating no real increase in costs (or activity) from patient-end services despite the increase in telehealth (*p* = 0.096). The insignificant changes in overall MBS costs resulted from the reduction in cost per telehealth specialist consultation, which was reduced after the COVID-19 MBS telehealth funding changes. This equivalence in overall costs before and after March 2020 is likely due to the reduction of telehealth reimbursement from 150% of the in-person reimbursement to parity payment for comparable in-person specialist consultations, as well as the reduction in patient-end services relative to the increase in telehealth.

## Discussion

The COVID-19 MBS telehealth funding changes introduced by the Australian government in March 2020 enabled all patients to receive continued specialist care during COVID-19. The pandemic was a catalyst for the near-immediate increase in specialist telehealth activity observed, facilitated by relaxing eligibility restrictions for telehealth reimbursement. Overall, specialist activity did not increase after the onset of COVID-19, demonstrating that telehealth was mainly used to substitute in-person care. However, telephone consultations accounted for a large proportion of telehealth use after the onset of COVID-19, with minimal video consultations. As a whole, the costs to the MBS for specialist consultations did not differ significantly after COVID-19 telehealth funding was introduced. Therefore, the provision of specialist care from the MBS perspective resulted in increased cost efficiency when telehealth was used. This was because a higher number of telehealth consultations were delivered for the same cost to the MBS, as telehealth reimbursement was reduced from 150% of the in-person reimbursement to parity payment for comparable in-person consultations.

The findings from this study demonstrate the influence of COVID-19 and MBS funding on specialist activity and costs. The high use of telephone over video consultations has been observed consistently across the primary care sector (GP consultations) in Australia.^12^ However, specialist and allied health consultations seem more likely to be delivered by video consultations than those delivered by GPs. Increases in telehealth during COVID-19, particularly telephone consultations, have also been observed in other countries such as the United States and Canada.^14–16^ Although telehealth has been a key strategy in mitigating potential COVID-19 transmission, this does not negate the need to assess the quality of care. Research into telehealth benefits has long been recognised,^17–19^ along with a demonstration of patient and clinician telehealth satisfaction,^20,21^ and no negative effects on clinical effectiveness or mortality outcomes.^22,23^ However, an investigation into the differences in effectiveness between telephone and video consultations is still required. Telehealth is not intended to completely replace in-person care, although COVID-19 created a natural experiment that has revealed circumstances where patients do not necessarily require a physical consultation. These observed changes in specialist activity and costs could be used in the development of alternative models of specialist care and to help inform future policy decisions.

Interestingly, the insignificant change in overall specialist activity after the onset of COVID-19 differs from the increase in overall activity observed in the primary care sector.^3^ Unlike specialist care, the average monthly number of GP consultations increased after the introduction of COVID-19 MBS telehealth funding,^3,12^ signifying the implementation of new telehealth services rather than telehealth acting as a substitution for in-person services. This increase in telehealth consultations by primary care providers resulted in an increase in MBS costs.^3^ However, for specialists, costs to the MBS remained the same, primarily due to telehealth substitution of in-person care, along with the decrease in telehealth reimbursement value from 150% to at parity payment. There was no significant change in patient-end services since the onset of COVID-19, which did not align with the overall increase in telehealth. This signifies a divergence from the historical service delivery model where a clinician is present at both ends of the video consultation.

Given the changes to MBS telehealth funding during COVID-19, and the surge in telephone consultations, telehealth funding reforms are of high interest in Australia and globally. To help guide reimbursement decisions, assessing the cost-effectiveness of telephone and video consultations is essential. Identifying the most appropriate clinical situations to use telephone and videoconference modes across diverse specialist disciplines will be important in guiding future funding decisions. Since the onset of COVID-19, Canada and the United States also expanded telehealth coverage to all patients and reimbursed telephone and video consultations.^16^ Telehealth funding policies are still being optimised, particularly, in regard to the relative reimbursement amount for telephone and video consultations compared to in-person consultations. The findings from this study have shown that MBS overall costs for specialist activity have not changed substantially despite the increase in telehealth consultations, which is different from that observed in primary care. This highlights the importance of ensuring the sustainability of telehealth services and ensuring efficient allocation of MBS resources.

### Limitations

Until the impacts of COVID-19 have fully unfolded, the sustained rate of telehealth specialist consultations is yet to be revealed. This study described specialist activity after the onset of COVID-19; however, ongoing analysis will be required in a post-pandemic setting. This will enable further examination of other key drivers (e.g. clinician willingness or funding reforms) that may have influenced increases in telehealth. Investigation into the differences between telephone and videoconference modes will be required to help guide future funding decisions. This study used data that analysed MBS activity and costs, therefore, an investigation into the clinical effectiveness of telehealth could not be explored and should be a focus area for future research. This study analysed MBS-claimed specialist consultations, therefore public hospital specialist services and other private specialist care were not represented within the data.

## Conclusion

The introduction of COVID-19 MBS telehealth funding enabled patients to receive continued specialist care in Australia during lockdown and while in isolation. The removal of geographical restrictions for telehealth funding also meant that all of the Australian population had access to subsidised telehealth consultations. Telehealth dramatically increased during COVID-19, with high use of telephone compared to video consultations. After the onset of COVID-19, the cost per telehealth specialist consultation was reduced from 150% of the in-person reimbursement to parity payment for comparable in-person consultations. Telehealth consultations substituted in-person specialist care during COVID-19 and fewer consultations had an associated patient-end claim. This resulted in increased cost efficiency to the MBS when telehealth was used, as a higher number of specialist consultations were delivered for the same cost.
